# Xuebijing Ameliorates Sepsis-Induced Lung Injury by Downregulating HMGB1 and RAGE Expressions in Mice

**DOI:** 10.1155/2015/860259

**Published:** 2015-03-02

**Authors:** Qiao Wang, Xin Wu, Xiaowen Tong, Zhiling Zhang, Bing Xu, Wugang Zhou

**Affiliations:** Department of Emergency, Shanghai Ninth People's Hospital, School of Medicine, Shanghai Jiao Tong University, Room 210, Building 10, No. 639, Zhi Zao Ju Road, Huang Pu District, Shanghai 200011, China

## Abstract

Xuebijing (XBJ) injection, a traditional Chinese medicine, has been reported as a promising approach in the treatment of sepsis in China. However, its actual molecular mechanisms in sepsis-induced lung injury are yet unknown. Therefore, this study aimed to investigate the beneficial effects of XBJ on inflammation and the underlying mechanisms in a model of caecal ligation and puncture-(CLP-) induced lung injury. The mice were divided into CLP group, CLP+XBJ group (XBJ, 4 mL/kg per 12 hours), and sham group. The molecular and histological examinations were performed on the lung, serum, and bronchoalveolar lavage (BAL) fluid samples of mice at the points of 6, 24, and 48 hours after CLP. The results show that XBJ reduces morphological destruction and neutrophil infiltration in the alveolar space and lung wet/dry weight ratio, which improves mortality of CLP-induced lung injury. Meanwhile, XBJ treatment downregulates high mobility group box protein 1 (HMGB1) and the receptor for advanced glycation end products (RAGE) expression, as well as neutrophil counts, production of IL-1*β*, IL-6, and TNF-*α* in the BAL fluids. In conclusion, these results indicate that XBJ may reduce the mortality through inhibiting proinflammatory cytokines secretion mediated by HMGB1/RAGE axis.

## 1. Introduction

Sepsis is characterized by a syndrome of systemic inflammation in response to infection, and it remains the leading and increasing cause of death in intensive care units [1, 2]. Sepsis causes multiple organ failure and acute lung injury (ALI) is a critical complication of sepsis [3]. Moreover, the mortality rate for patients with ALI ranges from 34% to 58% [4]. The unacceptably high mortality rate has inspired people to develop more molecular pathogenesis of ALI and improve therapeutic interventions.

High mobility group box protein 1 (HMGB1), released from necrotic and activated immune cells, acts as a proinflammatory cytokine into the extracellular milieu and has been implicated in the development of endotoxin-induced ALI as a late mediator [5, 6]. Evidences indicated that HMGB1 levels markedly increased during ALI in animals and humans [7, 8]. More importantly, the characteristics of HMGB1 provide a wider time frame for clinical intervention of neutralizing the inflammatory cascade, making this cytokine a more effective therapeutic target than other proinflammatory cytokines [9]. HMGB1 achieves cytokine-like properties, activating cellular signals such as NF-kB pathways, by interacting with the receptor for advanced glycation end products (RAGE) [10]. RAGE, mainly located in the alveolar type I cells, is a multiligand-binding receptor with an extracellular, transmembrane, and cytosolic domain [11, 12]. Similarly, in the murine model and the clinical trial of ALI, RAGE levels were not only elevated in the bronchoalveolar fluid and serum, but also closely related to the severity of lung injury [13].

Xuebijing (XBJ) injection is a traditional Chinese medicine, consisting of Carthami flos, Paeoniae Radix Rubra, Chuanxiong Rhizoma, Salviae miltiorrhizae, and Angelicae sinensis Radix [14]. Furthermore, XBJ has been widely applied to the clinical treatment of sepsis in China and approved by the State Food and Drug Administration (SFDA) of China [14–16]. Also, several studies showed that therapeutic effects were involved in the anti-inflammatory features of XBJ, resulting from promoting annexin A1 expression and inhibiting proinflammatory cytokines, such as interleukin-1*β* (IL-1*β*), IL-6, IL-8, IL-23, and tumor necrosis factor-*α* (TNF-*α*) [17–19].

Although the beneficial effects of XBJ have been widely investigated, the actual molecular mechanisms of XBJ are still unclear. Therefore, we used the animal model of caecal ligation and puncture (CLP), because CLP is the most widely used model and more closely mimics the clinical conditions of sepsis-induced lung injury [20, 21]. Moreover, the present study is designed to determine whether XBJ can suppress the development of sepsis-induced lung injury through inhibiting the inflammatory signaling pathway mediated by HMGB1 and RAGE, which plays an essential role in the pathological process of lung injury previously described.

## 2. Materials and Methods

### 2.1. Animals

Male C57BL/6 mice (10–12 weeks old; 20–25 g) were purchased from the Chinese Academy of Sciences and housed in a reversed 12 : 12-hour light-dark cycle in a temperature-controlled facility (21 ± 1°C) with free access to standard laboratory chow and tap water. The investigation conformed to National Institutes of Health Guidelines for the Care and Use of Laboratory Animals and was approved by the Committee on Ethics of Biomedicine Research (Shanghai Jiaotong University School of Medicine, Shanghai, China).

### 2.2. Animal Model of CLP-Induced Lung Injury during Sepsis

An animal model of CLP-induced lung injury during sepsis was performed as described previously [22, 23]. Mice were anesthetized with 50 mg/kg pentobarbital by intraperitoneal injection. A midline incision (approximately 1 cm) in abdominal wall was made to exteriorize the cecum. The cecum was then ligated 1 cm from the apex with 3-0 silk suture. The distal caecum was punctured through and through with a sterile 18-gauge needle. A small amount of luminal content was extruded into the peritoneal cavity. After the caecum was returned to the abdominal cavity, abdominal muscle and skin incisions were closed. All mice received fluid resuscitation with 30 mL/kg normal saline fluid injected subcutaneously after the surgery.

### 2.3. Experimental Design

Five minutes after CLP, mice were randomly divided into 2 treatment groups that received tail vein injection every 12 hours as follows: saline (CLP group, 4 mL/kg); XBJ (CLP+XBJ group, Chase Sun Pharmaceutical Co., Tianjin, China, 4 mL/kg). Besides, the normal control group (sham group) underwent sham surgery in which the cecums were exteriorized as described previously without ligation and puncture. To study the effect of CLP on the lung injury during sepsis, mice were sacrificed at the points of 6, 24, and 48 hours after CLP (*n* = 8 per time point).

### 2.4. Survival Rate

Five minutes after CLP, mice were treated with saline or XBJ every 12 hours as described above. The survival rate was observed for 168 hours (7 days). The CLP group and the CLP+XBJ group were composed of 30 animals. Besides, the sham group was composed of 6 animals.

### 2.5. Histopathological Analysis of Lung

The lung tissues were embedded in paraffin, cut into 4 *μ*m thick slices, and stained with classical hematoxylin and eosin (H & E). The lung injury score was performed by the method of semiquantitative assessment described previously [24, 25]. All histological specimens were coded and interpreted under a light microscope (Olympus, Tokyo, Japan) in a blinded manner.

### 2.6. Measurement of Wet/Dry Lung Weight Ratios

The water content of the lung, representing the severity of pulmonary edema, was measured by calculating the wet/dry weight ratio of lung tissues [26]. The left lung was cut and the wet weight was measured after water and blood staining on the lung tissue surface was absorbed with filter paper. Then, the same lung was dried at 80°C for 48 hours to obtain the dry weight, and the wet/dry ratio was calculated.

### 2.7. Differential Leukocyte Counts and Inflammatory Cytokines in the BAL Fluids

Mice were anesthetized, the tracheae were cannulated, and lungs were washed three times with 1.8 mL of phosphate-buffered saline (PBS) in total, and then bronchoalveolar lavage (BAL) fluids were gathered. Some BAL fluids were used to determine total leukocytes count immediately by a hemocytometer. Differential leukocytes counts were enumerated by counting at least 500 leukocytes under a light microscope using May-Grunwald-Giemsa staining.

The remaining collected BAL fluids were centrifuged at 1000 g for 10 min and the supernatant was collected and stored at −80°C for later TNF-*α*, IL-1*β*, and IL-6 levels measurement by enzyme-linked immunosorbent assays (ELISA) kits (Biosource International, Carlsbad, CA) as per the instruction of manufacturer.

### 2.8. HMGB1 Measurement in Serum

Blood samples (0.5 mL) were collected by cardiac puncture under anesthesia from mice. After centrifuging, the serum was stored at −80°C. The circulating HMGB1 levels were determined by ELISA (HMGB1 ELISA Kit, IBL Int. Corp., Toronto, Canada) according to the manufacturer's protocol.

### 2.9. Western Blot Analysis for HMGB1 and RAGE in Lung Tissues

The protein levels of HMGB1 and RAGE in lung tissues were measured by the western blot analysis as described previously [27, 28]. Briefly, soluble protein extracts (20 *μ*g) from the lung tissues were subject to 8% SDS-polyacrylamide gels and then transferred to polyvinyldifluoride (PVDF) membranes. After blocking in nonfat milk, the membranes were exposed to primary anti-HMGB1 antibody (1 : 1000 dilution; Abcam, Hong Kong, China) or anti-RAGE antibody (1 : 1000 dilution; Abcam, Hong Kong, China) overnight at 4°C. After incubation with horseradish peroxidase-linked anti-rabbit secondary antibodies, the proteins were detected with enhanced chemiluminescence reagent and finally exposed to X-ray films for the detection of target proteins.

### 2.10. Statistical Analysis

All data were expressed as means ± SEM values. Comparisons were performed by either Student's *t*-test, analysis of variance (ANOVA) followed by Bonferroni test for multiple comparisons, or the nonparametric Kruskal-Wallis test. The survival curves were drawn followed by Kaplan-Meier methods using the log-rank test. Statistical significance was indicated by *P* < 0.05.

## 3. Results

### 3.1. The Effect of XBJ on Reducing the Mortality of Mice with CLP-Induced Lung Injury during Sepsis

**Figure 1:**
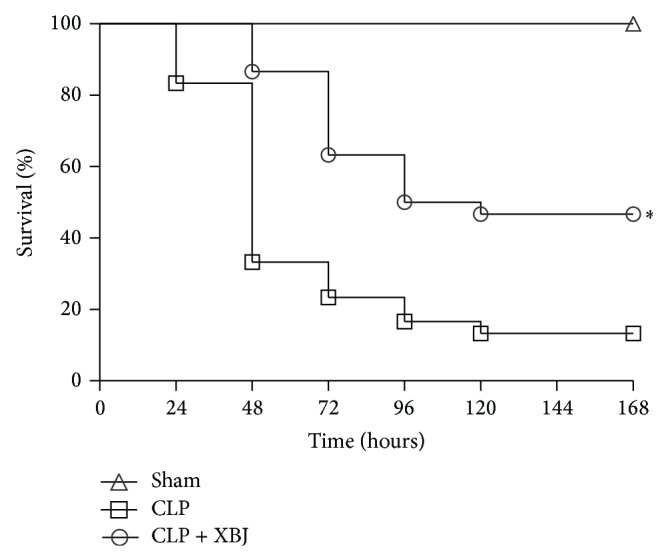
The Kaplan-Meier survival curve after CLP in mice treated with Xuebijing (XBJ). The survival rate was assessed for 168 hours. After CLP, mice received saline (CLP group, *n* = 30), XBJ (CLP+XBJ group, *n* = 30). The sham control group that underwent sham surgery (sham group, *n* = 6) were intravenously injected with saline. The group given XBJ had a significantly greater survival rate than the group given saline after CLP (*P* < 0.05). ^*^ *P* < 0.05 versus CLP group. CLP, caecal ligation and puncture.

First, we aimed to study therapeutic effects of XBJ in CLP-induced lung injury. As shown in Figure 1, mice in the CLP group exhibited a high mortality of 66.7% at 48 hours, 76.7% at 72 hours, 83.3% at 96 hours, and 86.7% at 120 hours, which was markedly reduced by XBJ treatment (13.3% mortality at 48 hours, 36.7% mortality at 72 hours, 50% mortality at 96 hours, and 53.3% mortality at 120 hours; *P* < 0.05). These results indicate that XBJ has beneficial effects on the mortality of CLP-induced lung injury.

### 3.2. XBJ Attenuated the Inflammation in the Lung on Histopathology

**Figure 2:**
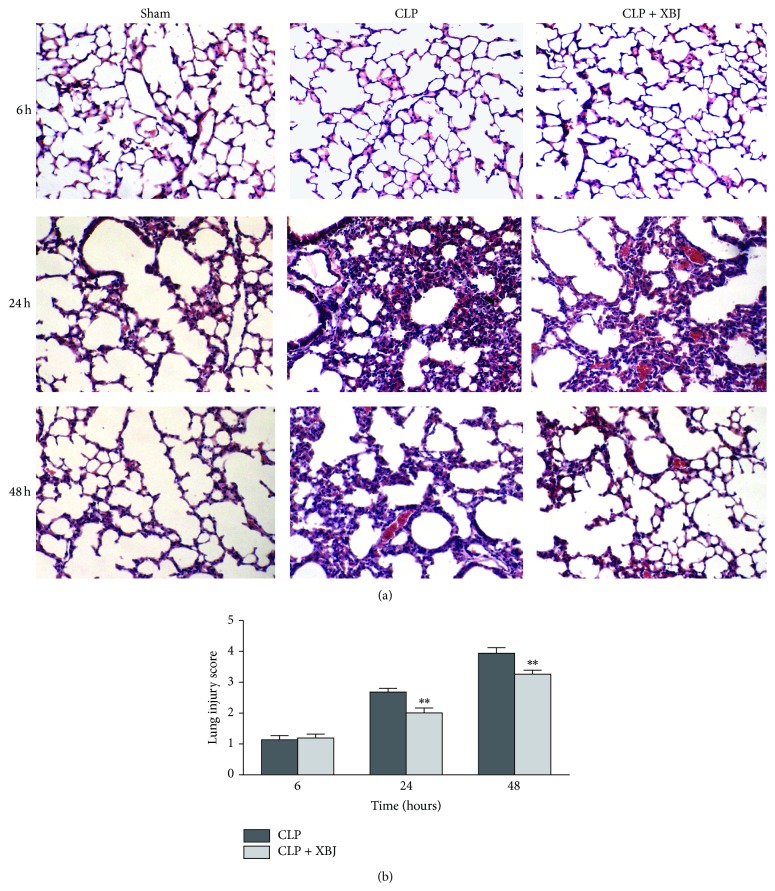
Xuebijing (XBJ) inhibits inflammation in the injured lung. (a) Representative histological lung sections from each group. Tissues were stained with hematoxylin and eosin (H & E). CLP-induced lung injury with morphological destruction and neutrophil infiltration at 6, 24, and 48 hours. These changes were significantly attenuated by treatment with XBJ (*n* = 8). Original magnification, ×100; HE, hematoxylin and eosin. (b) Lung injury score. The lung injury scores of inflammation on histology at 24 and 48 hours after CLP were significantly reduced by XBJ treatment. Data are expressed as means ± SEM for 8 mice per group. ^**^ *P* < 0.01 versus respective CLP group. CLP, caecal ligation and puncture.

To evaluate the anti-inflammatory effects of XBJ, the lungs were examined histologically at the points of 6, 24, and 48 hours after CLP by staining with H & E. At 6 hours, histopathology of the lung sections demonstrated an acute inflammatory response including interstitial edema and inflammatory cells infiltrate into the interstitium and alveolar spaces. With the time going by, the severity of acute inflammation in the interstitium and alveolar spaces was getting worse. And at 48 hours, histopathology of the lung sections showed the thickening of the alveolar wall, more infiltrating cells and hemorrhage throughout the lung tissue, and the destruction of alveolar structures. These changes were significantly attenuated after CLP mice were treated with XBJ (Figure 2(a)). Moreover, quantitative histology demonstrated that the lung injury scores of inflammation in the CLP+XBJ group were significantly lower than those in the CLP group at 24 and 48 hours after CLP (*P* < 0.01; Figure 2(b)). These findings suggest that XBJ reduces the mortality through histologically improving inflammatory responses in the lung.

### 3.3. XBJ Decreased Lung Wet/Dry Weight Ratios

**Figure 3:**
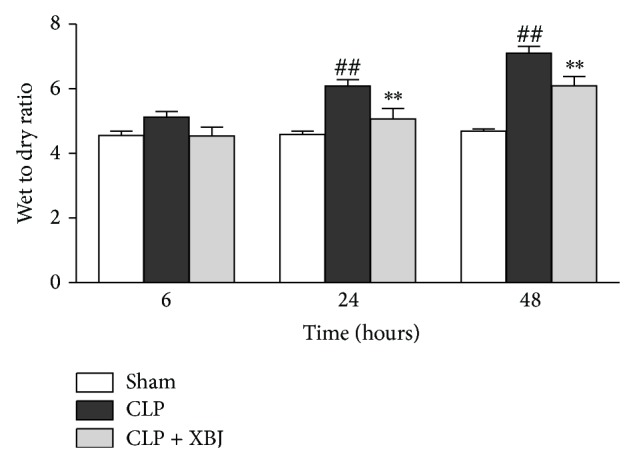
Xuebijing (XBJ) decreased lung wet/dry weight ratios. Data are expressed as means ± SEM for 8 mice per group. ^##^ *P* < 0.01 versus respective sham group; ^**^ *P* < 0.01 versus respective CLP group. CLP, caecal ligation and puncture.

The wet/dry weight ratios of lung tissues were determined for the purpose of detecting the improved pulmonary edema by XBJ treatment. As depicted in Figure 3, lung wet/dry weight ratios were evidently higher at 24 and 48 hours after CLP than those in the sham group (*P* < 0.01). XBJ treatment significantly reduced the lung wet/dry weight ratio at 24 and 48 hours after CLP compared with the CLP group (*P* < 0.01), suggesting that XBJ reduced the mortality of lung injury via ameliorating excessive lung water accumulation as well.

### 3.4. XBJ Ameliorated Inflammatory Cells and Cytokines in Bronchoalveolar Lavage (BAL) Fluids

**Figure 4:**
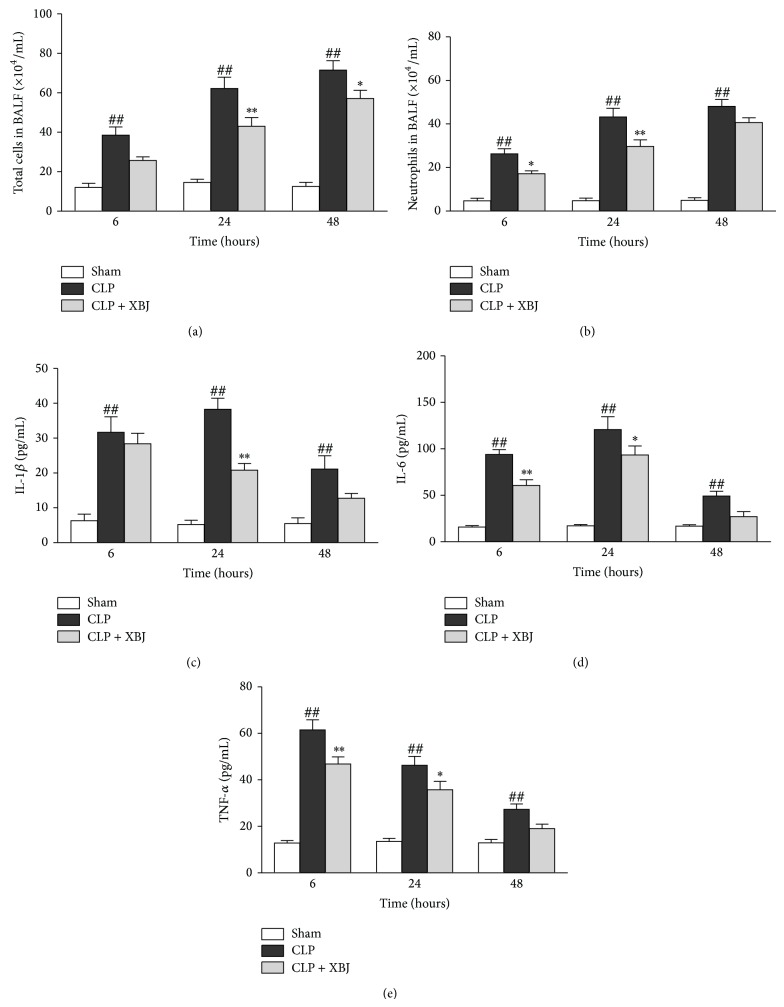
Inhibition effects of Xuebijing (XBJ) on neutrophils and inflammatory cytokines production in bronchoalveolar lavage (BAL) fluid of CLP-induced lung injury mice. (a) Total cell counts were performed. The BAL fluid was obtained from mice (*n* = 8) at 6, 24, and 48 hours after CLP. (b) The neutrophil counts were performed. The neutrophil counts were performed on a minimum of 500 cells to identify neutrophils. ((c)–(e)) Cytokines (IL-6, IL-1*β*, and TNF-*α*) in the BAL fluid were measured by ELISA at 6, 24, and 48 hours after CLP. Data are expressed as means ± SEM for 8 mice per group. ^##^ *P* < 0.01 versus respective sham group; ^*^ *P* < 0.05 versus respective CLP group; ^**^ *P* < 0.01 versus respective CLP group. CLP, caecal ligation and puncture.

Inflammatory cell counts and cytokines in BAL fluids were determined to evaluate the effects of XBJ on neutrophil accumulation into the alveolar space. Figures 4(a) and 4(b) illustrated that both total cells counts and neutrophils counts in BAL fluid significantly increased after CLP compared with those that underwent sham surgery (*P* < 0.01). XBJ treatment significantly decreased total cell counts in BAL fluid at 24 hours (*P* < 0.01) and 48 hours (*P* < 0.05) after CLP compared with the CLP group (Figure 4(a)). Moreover, mice treated with XBJ exhibited significantly lower numbers of neutrophils in BAL fluid at 6 hours (*P* < 0.05) and 24 hours (*P* < 0.01) than those given saline after CLP (Figure 4(b)).

As shown in Figures 4(c)–4(e), CLP administration induced significantly higher levels of IL-1*β*, IL-6, and TNF-*α* in the BAL fluid (*P* < 0.01 versus sham group). XBJ treatment significantly reduced the levels of IL-1*β* in BAL fluid at 24 hours after CLP compared with the CLP group (*P* < 0.01; Figure 4(c)). Moreover, IL-6 and TNF-*α* in BAL fluid of mice treated with XBJ obviously decreased at 6 hours (*P* < 0.01; Figures 4(d) and 4(e)) and 24 hours (*P* < 0.05; Figures 4(d) and 4(e)).

These results indicate that XBJ inhibits neutrophil infiltration and proinflammatory cytokines production.

### 3.5. Inhibition of HMGB1 and RAGE Expression from XBJ in CLP-Induced Lung Injury

In demonstrating the reduction in inflammation by XBJ, we investigated potential molecular mechanisms mediating the effect, such as HMGB1 and RAGE, which recently have been proved to act as an important part to the development of acute lung injury.

**Figure 5:**
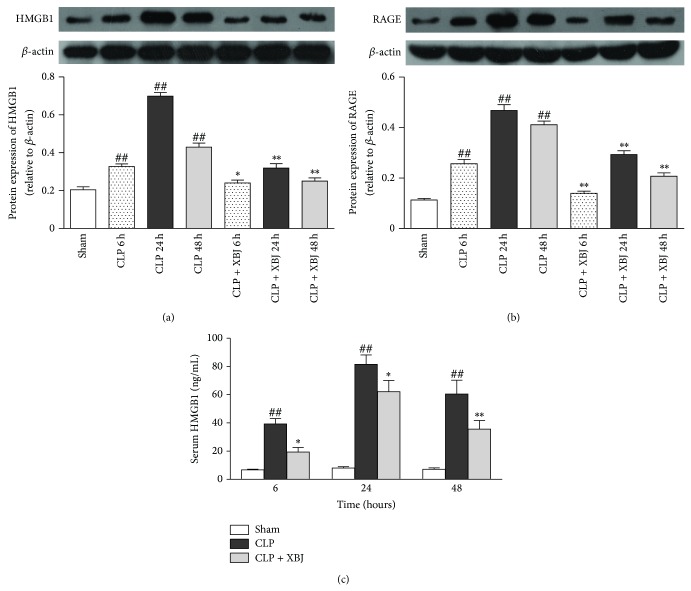
Inhibition of Xuebijing (XBJ) on HMGB1 and RAGE expression. (a) Western blot analysis of HMGB1 in the lungs. (b) Western blot analysis of RAGE in the lungs. (a)-(b) Representative western blot image (upper panel) and quantification (lower panel) analysis are shown. (c) HMGB1 in serum was measured by ELISA at 6, 24, and 48 hours after CLP. Data are expressed as means ± SEM for 8 mice per group. ^##^ *P* < 0.01 versus respective sham group; ^*^ *P* < 0.05 versus respective CLP group; ^**^ *P* < 0.01 versus respective CLP group. CLP, caecal ligation and puncture. HMGB1, high mobility group box protein 1. RAGE, receptor for advanced glycation end products.

As shown in Figure 5(a), HMGB1 expression in the lung significantly increased at 6, 24, and 48 hours after CLP (*P* < 0.01). In addition, dynamic modulations of HMGB1 showed that HMGB1, arising at 6 hours, reached the top level at 24 hours and then dropped slightly at 48 hours after CLP. After the septic mice were treated with XBJ, the levels of HMGB1 significantly decreased in lung tissues at 6, 24, and 48 hours compared with those of CLP group (*P* < 0.05 for 6 hours, *P* < 0.01 for 24 and 48 hours).

Similarly, Figure 5(c) showed that the circulating serum level of HMGB1 significantly arose at 6, 24, and 48 hours after CLP surgery (*P* < 0.01), with the enhancement being most significant at 24 hours. Also, treatment with XBJ led to an apparent reduction at three different time points as compared with the respective CLP group (*P* < 0.05 for 6 hours and 24 hours, *P* < 0.01 for 48 hours).

In the meantime, dynamic modulations of RAGE expressions in the lungs with or without XBJ treatment were also measured at 6, 24, and 48 hours after CLP. As shown in Figure 5(b), the levels of RAGE in CLP group obviously went up after CLP (*P* < 0.01), whose time-concentration curve was in line with that of HMGB1. Moreover, XBJ treatment could notably reduce RAGE expression in lung tissues compared with the CLP group in all the three time periods (*P* < 0.01).

These results suggest that XBJ prevents HMGB1 and RAGE expression and interaction, which could further induce and aggravate the inflammation in the lung.

## 4. Discussion

In the present study, we investigate the underlying mechanisms of anti-inflammatory effect from XBJ and observe the effect of XBJ on HMGB1 and RAGE expression during CLP-induced lung injury. The results of this study have demonstrated that XBJ reduces mortality of mice with CLP-induced lung injury and mitigates inflammatory responses in the alveolar space, such as neutrophil infiltration and edema formation. Meanwhile, XBJ treatments on lung injury in mice downregulate HMGB1 and RAGE expression in the lung, as well as production of IL-1*β*, IL-6, and TNF-*α* in the BAL fluid. These findings suggest that XBJ could be protective against the development of lung injury by inhibiting HMGB1 and its receptor-RAGE interaction and subsequently reducing inflammatory cytokines secretion.

XBJ is a herbal prescription composed of five species of medicinal plants, whose constituents were identified by high-performance liquid chromatography (HPLC) system in the previous studies [14, 18], and shows satisfactory anti-inflammatory effects in the animal and clinical experiment [29, 30]. Consistent with previous reports, the present study shows that CLP-induced lung injury resulted in 66.7%, 76.7%, 83.3%, and 86.7% mortality at 48, 72, 96, and 120 hours, respectively, whereas the survival rates of CLP+XBJ treatment group were significantly higher than CLP group at the above different times.

As known to all, inflammation plays a key role in the pathogenesis and development of the sepsis-induced lung injury. The present study shows that XBJ treatment in lung injury obviously decreased morphological destruction and neutrophil accumulation into the alveolar space in the histopathology of the lung. Additionally, XBJ treatment significantly reduced wet/dry weight ratio, representing the water gain of pulmonary edema, during CLP-induced lung injury in mice, while it was well known that excessive water exacerbated respiratory failure. These results prove that XBJ could reduce the mortality of lung injury through ameliorating the inflammatory responses and pulmonary edema in the lung.

Although XBJ has a beneficial effect on lung injury and some studies have been proposed for the mechanisms, the molecular mechanisms of the protection from XBJ remain unclear. Recent investigations have demonstrated that HMGB1 and its pertinent receptor-RAGE have increasingly become important prognostic indices and potential therapeutic targets in the development of sepsis-induced lung injury. Ueno et al. observed that the concentrations of HMGB1 significantly increased in plasma and BAL fluids of mice with lung injury and intratracheal instillation of recombinant HMGB1 per se caused lung injury in mice [8]. Additionally, Salviae miltiorrhizae and Angelicae sinensis, which are two major components of the Xuebijing, have been proven to be protective against sepsis by inhibiting HMGB1 release [31, 32]. Consistent with the previous studies, the present study shows that HMGB1 expressions in the serum and lungs of CLP-induced lung injury group significantly increased and reached the top level at 24 h period. Nevertheless, HMGB1 in the serum and lung tissues of XBJ treatment group significantly reduced at 6, 24, and 48 hours after CLP, respectively. Subsequently, dynamic modulations of RAGE expressions treated with or without XBJ in the lung of septic mice are also determined in the present study. Some studies indicate that RAGE is the major receptor for the proinflammatory activity of HMGB1 and RAGE activation through HMGB1 has been involved in mediating sepsis [33–35]. In accordance with the previous reports, our data also showed that dynamic modulations of HMGB1 were similar to those of RAGE in Figure 5. Furthermore, the present study showed that CLP administration induced a significant increase in the RAGE expression at the three time points similar to dynamic modulations of HMGB1, which is in line with the previous study illustrating that RAGE expressions were markedly stepped up in human and animal septic objects [13]. After the treatment with XBJ, the levels of RAGE also decreased notably in lung tissues of septic mice. These findings suggest that XBJ could inhibit HMGB1 and RAGE expression and interaction, which could lead to activation of downstream inflammatory cytokines.

It has been demonstrated that increased production of proinflammatory cytokines and neutrophil accumulation could cause manifested inflammation, and Smit et al. observed that upregulated HMGB1 induced these phenomena via activation of RAGE in aspiration lung injury [36]. Therefore, to assess whether XBJ treatment can reduce inflammatory cells counts and cytokines through reduced HMGB1 and RAGE expressions in the lung, differential cell counts and TNF-*α*, IL-1*β*, and IL-6 levels were simultaneously investigated in the BAL fluids of the mice treated with or without XBJ at 6, 24, and 48 hours after CLP. We found that both total cells and neutrophils counts increased in BAL fluid after CLP, whereas XBJ treatment significantly decreased them. Correspondingly, upregulated TNF-*α*, IL-1*β*, and IL-6 levels after CLP were obviously reduced by XBJ treatment as well. Our data are in accordance with the study of He et al. [17], showing that proinflammatory cytokines in rat serum and neutrophil infiltration in the lung tissues of CLP+XBJ group were remarkably lower than those of CLP group. Additionally, consistent with the previous study showing that reduced HMGB1 and RAGE interaction suppressed TNF-*α*, IL-1*β*, and IL-6 expression [37], dynamic modulations of IL-1*β* and IL-6 levels at three time points after CLP were similar to those of HMGB1 and RAGE expressions in the present study and levels of HMGB1, RAGE, IL-1*β*, and IL-6 all dropped notably by XBJ treatment, which implicated that XBJ might mitigate neutrophils infiltration and cytokines releases, resulting from inhibiting HMGB1 and RAGE expression.

In summary, the present study proves that XBJ has beneficial effects on the lung injury induced by CLP. Moreover, the protective effects of XBJ in the development of CLP-induced lung injury during sepsis may be relevant to the modulation of cytokines-mediated inflammation in the lung via downregulation of HMGB1 and RAGE expressions.

## 5. Conclusions

In conclusion, XBJ treatment ameliorates the inflammatory responses in the lung, thereby reducing the mortality of CLP-induced lung injury. Furthermore, our novel finding indicates that XBJ may inhibit proinflammatory cytokines releases mediated by HMGB1/RAGE axis.
